# Electrospun Carbon Nanofibers with Embedded Co-Ceria Nanoparticles for Efficient Hydrogen Evolution and Overall Water Splitting

**DOI:** 10.3390/ma13040856

**Published:** 2020-02-13

**Authors:** Seongwon Woo, Jooyoung Lee, Dong Sub Lee, Jung Kyu Kim, Byungkwon Lim

**Affiliations:** 1 School of Advanced Materials Science and Engineering, Sungkyunkwan University (SKKU), Suwon 16419, Korea; wsw0601@skku.edu (S.W.); ljy5424@skku.edu (J.L.); leedongsub92@gmail.com (D.S.L.); 2 School of Chemical Engineering, Sungkyunkwan University (SKKU), Suwon 16419, Korea

**Keywords:** electrospinning, carbon nanofiber support, overall water splitting, transition-metal-based electrocatalysts

## Abstract

In this study, simple electrospinning combined with pyrolysis were used to fabricate transition-metal-based-nanoparticle-incorporated carbon nanofiber (CNF) electrocatalysts for a high-efficiency hydrogen evolution reaction (HER) and overall water splitting. Co-CeO_2_ nanoparticle-incorporated carbon nanofibers (Co-CeO_2_@CNF) exhibit an outstanding electrocatalytic HER performance with an overpotential and Tafel slope of 92 mV and 54 mV/dec, respectively. For the counterpart, electrolysis, we incorporate the widely used Ni_2_Fe catalyst with a high oxygen evolution reaction (OER) activity into the carbon nanofiber (Ni_2_Fe@CNF). To evaluate their electrochemical properties for the overall water splitting, Co-CeO_2_@CNF and Ni_2_Fe@CNF were used as the HER and OER electrocatalysts in an alkaline electrolyzer. With the paired Co-CeO_2_@CNF and Ni_2_Fe@CNF electrodes, an overall water splitting current density of 10 mA/cm^2^ was achieved by applying 1.587 V across the electrodes with a remarkably lower overpotential of 257 mV compared to that of an electrolyzer comprised of Pt/C and IrO_2_ electrodes (400 mV). Owing to the conformal incorporation of nanoparticles into the CNF, the electrocatalysts exhibit significant long-term durability over 70 h of overall water splitting. This study provides rational designs of catalysts with high electrochemical catalytic activity and durability to achieve overall water splitting.

## 1. Introduction

The fabrication of high-efficiency catalysts with long-term durability through a cost-effective process is essential for the sustainable production of hydrogen (H_2_) [1,2]. Electro-chemical water splitting has been considered as a promising eco-friendly process that avoids the emission of greenhouse gases or harmful components (i.e., CO_2_ or NO_x_) [2,3]. In recent decades, several studies have been performed to replace noble-metal-based electrocatalysts with earth-abundant nonnoble metals and highly active materials such as transition-metal alloys, oxides, carbides, nitrides, and phosphides [4,5,6,7,8,9,10,11,12,13,14,15]. However, various issues need to be addressed to achieve high-efficiency overall water electrolysis. Such issues include the poor electrochemical catalytic kinetics with very high overpotentials on both electrocatalysts for the simultaneous hydrogen evolution reaction (HER) and oxygen evolution reaction (OER) [4,16]. Moreover, several durability issues exist regarding electrocatalysts during electrolysis. Even commercialized Pt/C and Pt alloys suffer from degradation in the electrolyte [17]. To improve the long-term stabilities of the catalysts, highly conductive carbon-based nanomaterials such as carbon nanotubes, reduced graphene oxide, and carbon nanofibers (CNFs) have been utilized as support or binding materials [18,19,20]. However, conformal incorporations of nanostructured catalysts into nanocarbon-based supports with a systemic formation should be realized to simultaneously ensure the sufficient electrochemical activity and high conductance of the carriers [20,21]. Therefore, the rational design of electrocatalysts, considering the process cost, catalytic activity, and long-term durability, is required to achieve high-efficiency overall water splitting for the production of clean H_2_. Recently, cost-effective electrospun nanofibers with a high conductivity and a large specific surface area have attracted considerable attention for electrochemical HER [22,23,24]. The highly conductive nanofibers obtained by electrospinning can be an excellent support for electrocatalysts owing to their outstanding mechanical strength, flexibility, and charge-transport properties [24,25,26].

In this study, simple electrospinning combined with pyrolysis were utilized to fabricate transition-metal-incorporated CNF electrocatalysts for efficient HER and overall water splitting. Cobalt-incorporated ceria (CeO_2_) nanoparticles on a conductive carbon nanofiber (Co-CeO_2_@CNF) were fabricated for HER. Owing to the abundant defect sites originating from oxygen vacancies and the favorable conversion of oxidation states between Ce^3+^ and Ce^4+^, CeO_2_ can be considered as an excellent support for cobalt to achieve increased electrochemical activity and good stability [21,27,28,29,30]. Co-CeO_2_ nanoparticles with an atomic Co:Ce ratio of 1:1 were homogeneously dispersed on the CNF supports, which provided outstanding electrocatalytic performance. For the counterpart, water electrolysis, the widely used Ni_2_Fe catalyst for OER was incorporated into the CNF. To evaluate their electrochemical properties for the overall water splitting, Co-CeO_2_@CNF and Ni_2_Fe@CNF were used as the HER and OER electrocatalysts in an alkaline electrolyzer, respectively. Their performances were compared with those of an electrolyzer fabricated by pairing Pt/C and IrO_2_ electrodes. With the paired Co-CeO_2_@CNF and Ni_2_Fe@CNF electrodes, an overall water splitting current density of 10 mA/cm^2^ was achieved by applying 1.587 V across the electrodes with lower overpotential of 257 mV. Owing to the conformal incorporation of nanoparticles into the CNF supports, the electrocatalysts exhibited significant long-term durability over 70 h of operation.

## 2. Materials and Methods

### 2.1. Synthesis of Electrocatalysts

For the typical electrospinning synthesis, 1.0 g of polyacrylonitrile (PAN, average *M*_w_ = 150,000, Sigma-Aldrich, Missouri, USA) was initially dissolved in N,N-dimethylformamide (Deajung) solvent (10 mL) under vigorous stirring for 6 h. To fabricate Co-CeO_2_@CNF for the HER, 4 mmol of Co(acac)_2_·4H_2_O and 4 mmol of Ce(NO_3_)_3_·6H_2_O were introduced into the PNA solution under rapid stirring for another 12 h. The resultant solution was loaded into a syringe with a 22-gauge needle which was electrically connected to a high-voltage power supply. The applied potential between the needle and fiber collector (aluminum foil) was 27 kV. Then, the electrospinning was conducted with a flow rate of 1.5 mL/h. For the fabrication of the OER catalyst, 2 mmol of Ni(NO_3_)_2_·4H_2_O and 1 mmol of Fe(acac)_3_·6H_2_O were dissolved into the PAN solution under stirring at 500 rpm for another 12 h. The metal-precursor-dissolved solution was loaded into a syringe with a 27-gauge needle and electrospun with the flow rate of 0.8 mL/h and applied voltage of 15 kV. The distance between the needle tip and fiber collector was 20 cm. After the electrospinning, the collected fiber films were stabilized in air at 280 °C for 3 h, then post thermal treatment was conducted at 900 °C for 5 h under a N_2_ atmosphere to obtain Co-CeO_2_@CNF. 

### 2.2. Material Characterization

To investigate the morphological properties of the as-fabricated electrocatalysts, scanning electron microscopy (SEM) and transmission electron microscopy (TEM) images were acquired using a JSM-7600F (Jeol Ltd., Tokyo, Japan) at the MEMS·Sensor Platform Center of Sungkyunkwan University (SKKU) and a JEM-2100F microscope (Jeol Ltd., Tokyo, Japan) operated at 200 kV, respectively. The crystalline properties of the sample powders were characterized by powder X-ray diffraction (XRD). The obtained patterns were further investigated by using Joint Committee on Powder Diffraction Standards (JCPDS, v2.1, ICDD, Pennsylvania, USA) Card No. For XRD characterization, a D8-Advances (Bruker AXS, Karlsruhe, Germany) diffractometer with a Cu K_α_ radiation source (*λ* = 0.15418 nm) was used. X-ray photo-electron spectroscopy (XPS) was performed using an ESCA2000 (VG Microtech, East Grinstead, UK).

### 2.3. Electrochemical Measurements

For the fabrication of the electrodes, we prepared Ketjen black as a conductive agent, polyvinylidene fluoride as a binder and a mixture of a catalyst in a weight ratio of 20:10:70. These components were dissolved in N-methyl-2-pyrrolidone. Then, the sample slurry was coated onto a carbon paper substrate (1 × 1 cm^2^) and a vacuum dry was conducted overnight at 80 °C. Polarization and cyclic voltammetry (CV) curves were obtained using a CHI600D electrochemical analyzer (CH instrument) in a 1-M KOH solution. For the three-electrode configuration, Hg/HgO and Pt wire were used as reference and counter electrodes, respectively. Chrono-potentiometric curves were obtained using a WBCS-3000 (Xeno Co.) in a 1-M KOH solution. Electrochemical impedance spectroscopy (EIS) measurements were performed at −1.1 V versus a reversible hydrogen electrode (RHE) (alternating-current excitation signal) in a frequency range of 100 kHz to 0.1 Hz. All potential values were converted to the RHE scale by using *E*_RHE_ = *E*_SCE_ + *E*^0^_SCE_ + 0.059 × pH, where *E*_SCE_ is the potential measured using a saturated calomel electrode (SCE) reference, and *E*^0^_SCE_ is the standard potential of the SCE at 25 °C (0.078 V), unless otherwise stated.

## 3. Results

**Figure 1:**
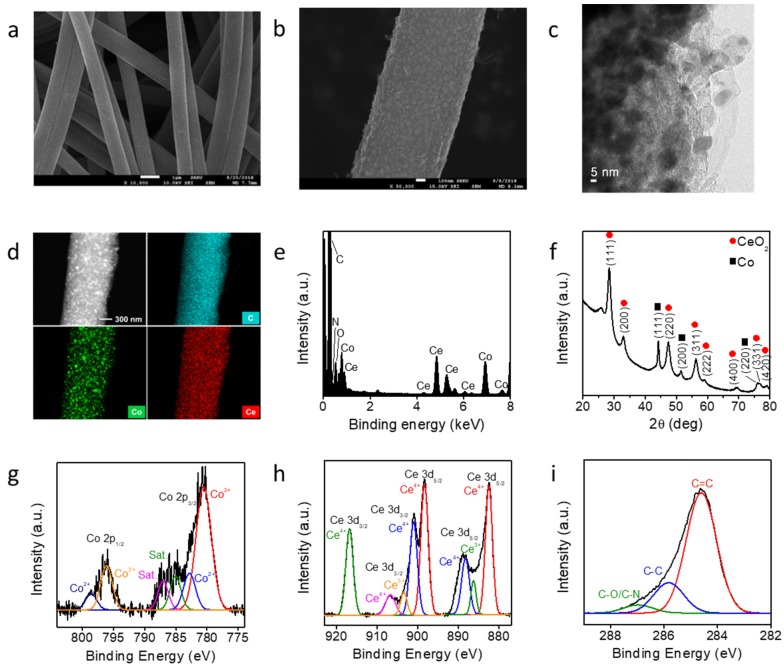
**Characterization of Co-CeO_2_@CNF.** (**a**,**b**) SEM and (**c**) TEM images, (**d**) scanning TEM image and EDS elemental maps, (**e**) EDS data, (**f**) XRD pattern, and (**g**–**i**) XPS analysis of Co-CeO_2_@CNF.

The Co-CeO_2_@CNF catalyst was fabricated by electrospinning and pyrolysis. A PAN solution containing metal precursors was electrospun on an aluminum current collector under an applied bias. The obtained polymer mat was transformed into a black Co-CeO_2_@CNF catalyst upon thermal treatment at 900 °C for 5 h in a tubular furnace under a N_2_ atmosphere. Figure 1a shows a typical SEM image of the synthesized Co-CeO_2_@CNF. A SEM image of a pristine CNF is shown in Figure S1 (Supplementary Information). The obtained nanofiber exhibited a one-dimensional structure with a diameter of approximately 840 nm. The higher-magnification SEM image shows that the surface of Co-CeO_2_@CNF was coarse, with numerous Co-CeO_2_ nanoparticles homogeneously dispersed in the nanofiber support (Figure 1b). The TEM image demonstrates the successful formation of Co-CeO_2_ nanoparticles on the surface of the CNF (Figure 1c). Co, Ce, C, N, and O elements were uniformly distributed in the CNF. An energy-dispersive X-ray spectroscopy (EDS) analysis shows an atomic ratio of Co:Ce of approximately 1:1 (Figure 1d,e, and Figure S2 in Supplementary Information). The XRD pattern of Co-CeO_2_@CNF shows three high peaks at 44.2°, 51.5°, and 75.8° corresponding to the (111), (200), and (220) planes of Co metal with a face-centered cubic (FCC) structure (JCPDS# 15-0806) respectively (Figure 1f). The formation of the FCC CeO_2_ (JCPDS# 81-0792) was confirmed by the diffraction peaks at 28.4, 32.9, 47.4, 56.3, 59.0, 69.4, 76.6, and 79.0° corresponding to the (111), (200), (220), (311), (222), (400), (331), and (420) planes, respectively. These results indicate that metal and oxide nanoparticles were formed in the CNF by electrospinning and pyrolysis. To understand the roles of the metal cations in the synthesis of Co-CeO_2_@CNF, bare Co@CNF and CeO_2_@CNF were synthesized by the same processes used for the synthesis of Co-CeO_2_@CNF but without the Co or Ce precursors. When the synthesis was performed without Ce(NO_3_)_3_ (the other conditions were identical to those for the sample in Figure S3a (Supplementary Information)), Co nanocrystals with a cubic structure were formed; this was confirmed by XRD (JCPDS Card No. 81-0792, Figure S3b in Supplementary Information). Without the Co(acac)_2_ precursor, CeO_2_ microparticles with a low-crystallinity cubic phase were obtained as the product (Figure S3c,d in Supplementary Information). These results indicate that both Co and Ce cations favored the formation of Co-CeO_2_ nanoparticles. Figure 1g–i depicts the XP spectra of Co 2*p*, Ce 3*d*, and O 1*s* respectively. The Co 2*p* core-level spectrum exhibited peaks at 780.5, 782.8, 785.0, and 787.0 eV, which were assigned as Co 2*p*_3/2_ Co^3+^, Co 2*p*_3/2_ Co^2+^, and satellite peaks, respectively. The peaks centered at 796.1 and 798.5 eV correspond to Co 2*p*_1/2_ Co^3+^ and Co 2*p*_1/2_ Co^2+^ respectively [31]. The Ce 3*d* core-level spectrum contained peaks associated with both Ce^3+^ and Ce^4+^ [32]. The electrons in the 4*f*^1^ orbital of Ce^3+^ could considerably influence the interaction between the ceria and the surrounding Co nanoparticles. Moreover, oxygen vacancies can be induced by the charge compensation of Ce^3+^ ions on the surface of CeO_2_ [1,31]. This can enhance the electrochemical water-splitting catalytic kinetics of the Co-CeO_2_-incorporated CNF. 

**Figure 2:**
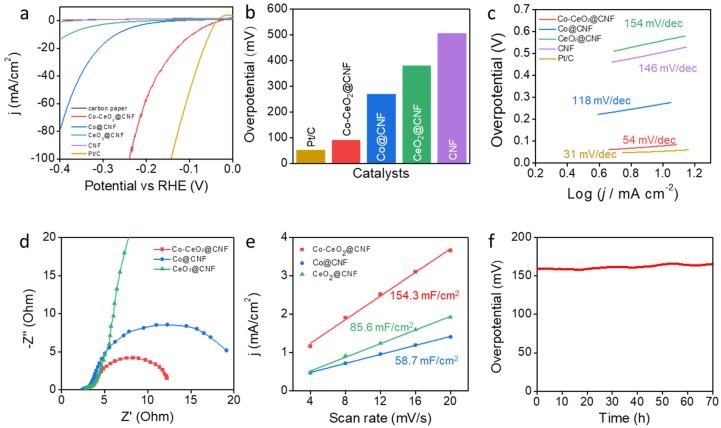
Electrochemical performances of the electrocatalysts for the HER in 1-M KOH. (**a**) HER polarization curves of the Co-CeO_2_@CNF, Co@CNF, CeO_2_@CNF, CNF, commercial Pt/C, and bare carbon paper electrodes recorded in the 1-M KOH solution at a scan rate of 1 mV/s from negative to positive potentials at room temperature. (**b**) Comparison of the HER overpotentials of the Co-CeO_2_@CNF, Co@CNF, CeO_2_@CNF, CNF, and commercial Pt/C electrodes in the 1-M KOH. (**c**) Tafel plots of the Co-CeO_2_@CNF, Co@CNF, CeO_2_@CNF, CNF, and commercial Pt/C electrodes for the HER. (**d**) EIS plots of the Co-CeO_2_@CNF, Co@CNF, and CeO_2_@CNF electrodes at an applied potential of −0.2 V vs. RHE. (**e**) Electrochemical active surface areas of the Co-CeO_2_@CNF, Co@CNF, and CeO_2_@CNF electrodes obtained by using the double-layer capacitances. (**f**) *iR*-uncorrected chronopotentiometry curve of the Co-CeO_2_@CNF electrode at a constant current density of −10 mA/cm^2^ over a period of 70 h.

We investigated the effects of the electrochemical catalytic properties of Co-CeO_2_@CNF on the HER and compared them with those of Co@CNF, CeO_2_@CNF, CNF, commercial Pt/C, and carbon paper electrodes. Figure 2a shows polarization curves for the HERs, including the bare carbon paper. The Co-CeO_2_@CNF electrode exhibited a significant overpotential of 92 mV at a current density of -10 mA/cm^2^ with the *iR* compensation; the corresponding values for the Co@CNF, CeO_2_@CNF, and bare CNF electrodes were 270, 381, and 506 mV, respectively, while that for the Pt/C electrode was 54 mV (Figure 2b). This value was also lower than previously reported Co and CeO_2_ based catalysts (Table S1). The Tafel slope of the Co-CeO_2_@CNF electrode was 54 mV/dec, considerably smaller than those of the Co@CNF (118 mV/dec), CeO_2_@CNF (154 mV/dec), and CNF (146 mV/dec) electrodes (Figure 2c). It was slightly larger than that of the Pt/C electrode (31 mV/dec) [33]. Figure 2d shows Nyquist plots obtained from an EIS measurement. Consistently with the HER activities, the charge-transfer resistance (*R*_ct_) of the Co-CeO_2_@CNF electrode was considerably smaller than those of the Co@CNF and CeO_2_@CNF electrodes. This is consistent with the low overpotential and high HER activity of Co-CeO_2_@CNF. Furthermore, the electrochemical active surface area was characterized by investigating the electrochemical double-layer capacitance (*C*_dl_) via CV (Figure S4 in Supplementary Information). The estimated *C*_dl_ of the Co-CeO_2_@CNF electrode was 154.3 mF/cm^2^, whereas those of the Co@CNF and CeO_2_@CNF electrodes were 58.7 and 85.6 mF/cm^2^, respectively (Figure 2e). The higher capacitance of Co-CeO_2_@CNF than that of Co@CNF and CeO_2_@CNF indicates that its unique porous structure and effective active sites contributed to its high HER performance. To investigate the long-term durability of Co-CeO_2_@CNF, its overpotential at the current density of −10 mA/cm^2^ was monitored over time without iR compensation (Figure 2f). Notably, no significant change in overpotential (159.3–166.1 mV) was observed over the 70 h of operation.

**Figure 3:**
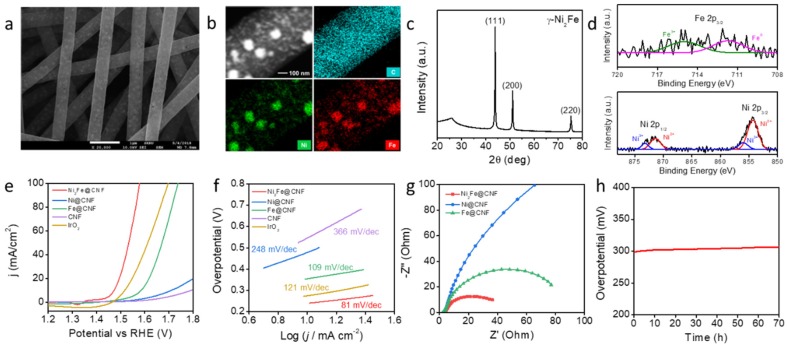
Ni_2_Fe@CNF for the OER electrode. (**a**) SEM images, (**b**) EDS elemental maps, (**c**) XRD pattern, and (**d**) XPS analysis of Ni_2_Fe@CNF. (**e**) Polarization curves of the Ni_2_Fe@CNF, Ni@CNF, Fe@CNF, CNF, commercial IrO_2_, and bare carbon paper electrodes recorded in a 1-M KOH solution at a scan rate of 1 mV/s from negative to positive potentials at room temperature. (**f**) Tafel plots of the Ni_2_Fe@CNF, Ni@CNF, Fe@CNF, CNF, and commercial IrO_2_ electrodes for the OER. (**g**) EIS plots of the Ni_2_Fe@CNF, Co@CNF, and CeO_2_@CNF electrodes at an applied potential of 1.53 V vs. RHE. (**h**) Chrono-potentiometry curve of the Ni_2_Fe@CNF electrode at a constant current density of 50 mA/cm^2^ over a period of 70 h.

For the counterpart, water electrolysis, Ni_2_Fe@CNF was used for the fabrication of the OER electrode by the same synthesis process as was used for Co-CeO_2_@CNF. The surface morphology of Ni_2_Fe@CNF was observed using SEM, as shown in Figure 3a. The resulting nanofibers had an average diameter of approximately 610 nm and an average particle size below 100 nm. The nanoparticles were encapsulated and well-embedded in the CNF. The EDS elemental maps in Figure 3b and Figure S5 in Supplementary Information show that Ni, Fe, C, N, and O elements were uniformly distributed in the CNFs. The XRD pattern of Ni_2_Fe@CNF shows diffraction peaks centered at 2*θ* values of 43.8°, 51.2°, and 75.3°, corresponding to (111), (200), and (220) reflections, respectively (Figure 3c). These results indicate that Ni_2_Fe metal nanoparticles were successfully formed in the CNF through electrospinning and pyrolysis. The Ni 2*p* core-level XP spectrum contained peaks associated with both Ni^2+^ (854.3 and 871.3 eV) and Ni^3+^ (856.0 and 873.2 eV). The Fe 2*p* core-level XP spectrum exhibited peaks corresponding to both Fe (707.1 eV) and Fe^3+^ (710.4 eV) (Figure 3d). Further, we evaluated the electrocatalytic performance of Ni_2_Fe@CNF for the OER and compared its performance with those of commercially utilized IrO_2_ nanoparticles with sizes of 30–150 nm and the control catalysts Ni@CNF, Fe@CNF, and bare CNF [33]. Figure 3e shows the OER polarization curves of five catalysts, including the commercial IrO_2_. With the *iR* compensation, the overpotential of the Ni_2_Fe@CNF electrode was 242 mV at a current density of 10 mA/cm^2^. The overpotentials of the Ni@CNF, Fe@CNF, CNF, and commercialized IrO_2_ catalysts were 478, 355, 561, and 284 mV, respectively. This OER performance was also similar or lower than that for previously reported Ni and Fe based catalysts (Table S2). The Tafel plot in Figure 3f shows the kinetics of the electrochemical OER on each catalyst. The Tafel slope of the Ni_2_Fe@CNF electrode was 81 mV/dec, considerably smaller than that of the Ni@CNF (248 mV/dec), Fe@CNF (109 mV/dec), and IrO_2_/NF (121 mV/dec) electrodes. The significantly low overpotential and small Tafel slope of Ni_2_Fe@CNF indicate its high OER efficiency. The Nyquist plots obtained from the EIS measurement reveal the charge-transfer resistances (*R*_ct_) during the electrochemical catalytic reactions of the Ni_2_Fe@CNF, Ni@CNF, and Fe@CNF electrodes. As shown in Figure 3g, the Ni_2_Fe@CNF electrode exhibited a considerably smaller *R*_ct_ of 36.5 Ω than the Ni@CNF (475.4 Ω) and Fe@CNF (77.0 Ω) electrodes did. The small *R*_ct_ reflects the favorable charge migration and desirable catalytic kinetics which led to the small Tafel slope. Moreover, the conformally embedded Ni_2_Fe nanoparticles in the CNF provided efficient electrical contacts and chemical stability of the composite, which also contributed to the superior stability [34]. To evaluate the long-term stabilities of the Ni_2_Fe@CNF electrode at large current densities, the overpotential for the OER was monitored at a constant current density of 50 mA/cm^2^ for 70 h. As shown in Figure 3h, no significant change in the potential required to maintain the current density of 50 mA/cm^2^ was observed in the period of 70 h. This demonstrates the excellent catalytic stability of the Ni_2_Fe@CNF electrode for the OER. 

**Figure 4:**
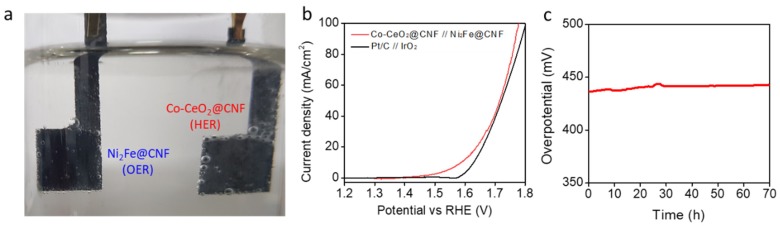
Electrochemical performances of the electrolyzer with Co-CeO_2_@CNF//Ni_2_Fe@CNF for overall water splitting. (**a**) Optical image of the Co-CeO_2_@//Ni_2_Fe@CNF electrolyzer. (**b**) Overall water splitting characteristics of the Co-CeO_2_@CNF//Ni_2_Fe@CNF and IrO_2_//(Pt/C) electrolyzers measured in a two-electrode configuration in the range of 1.8 to 1.2 V at a scan rate of 1 mV/s in a 1-M KOH solution. (**c**) *iR*-uncorrected chrono-potentiometric curve of the Co-CeO_2_@CNF//Ni_2_Fe@CNF electrolyzer at a steady-state current density of 10 mA/cm^2^ in a 1-M KOH solution.

For overall water splitting, we paired the Co-CeO_2_@CNF HER electrocatalyst with the high-performance (Figure S6 in Supplementary Information) Ni_2_Fe@CNF OER electrocatalyst (Figure S7 in Supplementary Information) in a 1-M KOH, to obtain an alkaline electrolyzer (Figure 4a). For the comparison, an electrolyzer was fabricated by pairing the Pt/C and IrO_2_ electrodes (Figure 4b). In the electrolyzer based on the Co-CeO_2_@CNF and Ni_2_Fe@CNF electrodes, an overall-water-splitting current density of 10 mA/cm^2^ was achieved by applying only 1.587 V across the electrodes with *iR* compensation. The lower overpotential of 257 mV was observed when that of the electrolyzer based on the Pt/C and IrO_2_ electrodes was 400 mV. In Figure 4c, the stability of the Co-CeO_2_@CNF and Ni_2_Fe@CNF electrodes was investigated by monitoring the applied potential values at a constant water-splitting current density of 10 mA/cm^2^. As shown in Figure 4c, the performance of the Co-CeO_2_@CNF and Ni_2_Fe@CNF electrodes was maintained over 70 h of chrono-potentiometry testing.

## 4. Conclusions

Simple electrospinning combined with pyrolysis were utilized to fabricate transition-metal-incorporated CNF electrocatalysts to achieve efficient overall water splitting. For the HER, Co-CeO_2_ nanoparticles with an Co:Ce atomic ratio of approximately 1:1 were homogeneously dispersed on CNF supports (Co-CeO_2_@CNF). Co-CeO_2_@CNF exhibited an outstanding electrocatalytic performance for the HER. Its overpotential and Tafel slope were 92 mV at a current density of −10 mA/cm^2^ and 54 mV/dec respectively. Ni_2_Fe@CNF was fabricated for the counterpart, water electrolysis. With the *iR* compensation, the overpotential of the Ni_2_Fe@CNF electrode was 242 mV at a current density of 10 mA/cm^2^. The Tafel slope of the Ni_2_Fe@CNF electrode was 81 mV/dec, considerably smaller than that of the commercialized IrO_2_/NF electrode (121 mV/dec). To evaluate their electrochemical properties for the overall water splitting, Co-CeO_2_@CNF and Ni_2_Fe@CNF were used as the HER and OER electrocatalysts in an alkaline electrolyzer, respectively. Their performances were compared with that of an electrolyzer fabricated by pairing Pt/C and IrO_2_ electrodes. With the paired Co-CeO_2_@CNF and Ni_2_Fe@CNF electrodes, an overall water splitting current density of 10 mA/cm^2^ was achieved by applying 1.587 V across the electrodes. The significantly lower overpotential of 257 mV was achieved when the overpotential of electrolyzer based on the Pt/C and IrO_2_ electrodes was 400 mV. Owing to the conformal incorporation of nanoparticles into the CNF supports, the electrocatalysts exhibited significant long-term durability. The performance of the Co-CeO_2_@CNF and Ni_2_Fe@CNF electrodes was maintained over 70 h of operation.

## Supplementary Materials

The supplementary materials are available online at https://www.mdpi.com/1996-1944/13/4/856/s1, Figure S1: SEM images of the pristine CNF fabricated via electrospinning and pyrolysis, Figure S2: EDS elemental mapping TEM images of the Co-CeO_2_@CNF representing as (a) O and (b) N, Figure S3: SEM images and XRD patterns of (a, b) Co@CNF and (c, d) CeO_2_@CNF, respectively, Figure S4: CV curves of (a)Co-CeO2@CNF, (b)Co@CNF, and (c)CeO2@CNF at various scan rates, Figure S5: EDS elemental mapping TEM images of the Ni2Fe@CNF representing as (a) O and (b) N, Figure S6: Polarization curves of Co-CeO_2_@CNF at various amount of Co-CeO_2_, Figure S7: Polarization curves of Ni_2_Fe@CNF at various amount of Ni_2_Fe, Table S1: HER performance compared to previously reported catalysts, Table S2: OER performance compared to previously reported catalysts.
