# Supplementary material for: Electrospun Carbon Nanofibers with Embedded Co-Ceria Nanoparticles for Efficient Hydrogen Evolution and Overall Water Splitting
Source: Materials (Basel). 2020 Feb 13;13(4):856. doi: 10.3390/ma13040856 (PMC7079610; doi:10.3390/ma13040856)
Supplement: Supplementary file 1 [file materials-13-00856-s001.pdf]

Supplementary Information

Electrospun Carbon Nanofibers with Embedded Co-Ceria Nanoparticles for Efficient Hydrogen Evolution and Overall Water Splitting

Seongwon Woo [1], Jooyoung Lee [1], Dong Sub Lee [1], Jung Kyu Kim [2,*], and Byungkwon Lim [1,*]

[1] School of Advanced Materials Science and Engineering, Sungkyunkwan University (SKKU), Suwon, 16419, Republic of Korea; wsw0601@skku.edu(S.W.), ljy5424@skku.edu(J.L.), leedongsub92@gmail.com(D.S.L.)
[2] School of Chemical Engineering, Sungkyunkwan University (SKKU), Suwon, 16419, Republic of Korea
* Correspondence: legkim@skku.edu (J.K.K); blim@skku.edu (B.L.)

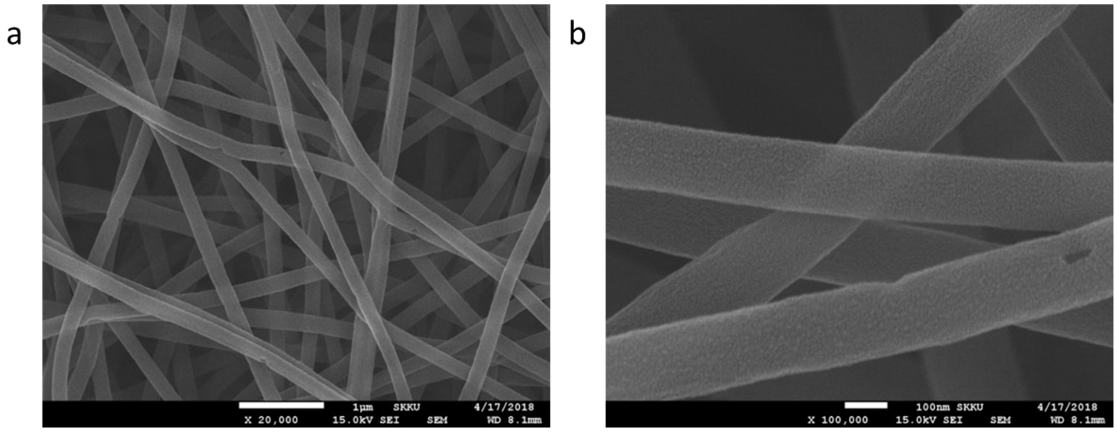

Figure S1. SEM images of the pristine CNF fabricated via electrospinning and pyrolysis.

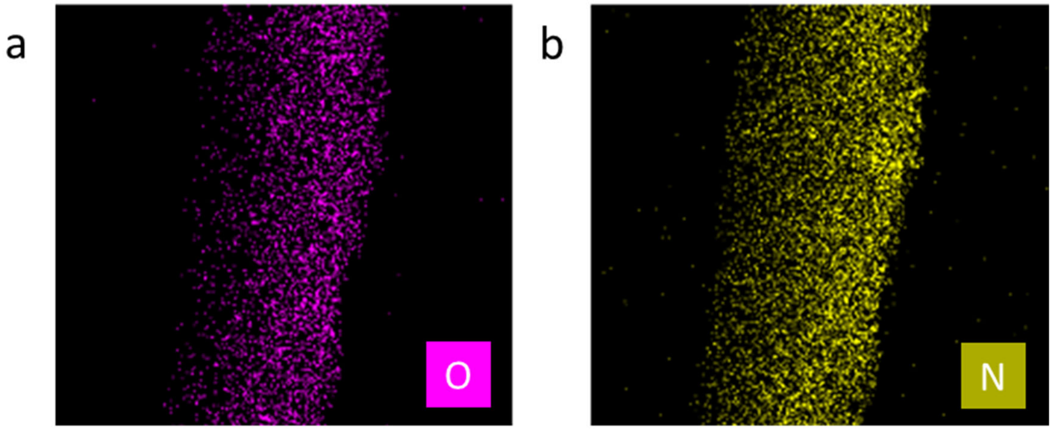

Figure S2. EDS elemental mapping TEM images of the Co-CeO_2@CNF representing as (**a**) O and (**b**) N.

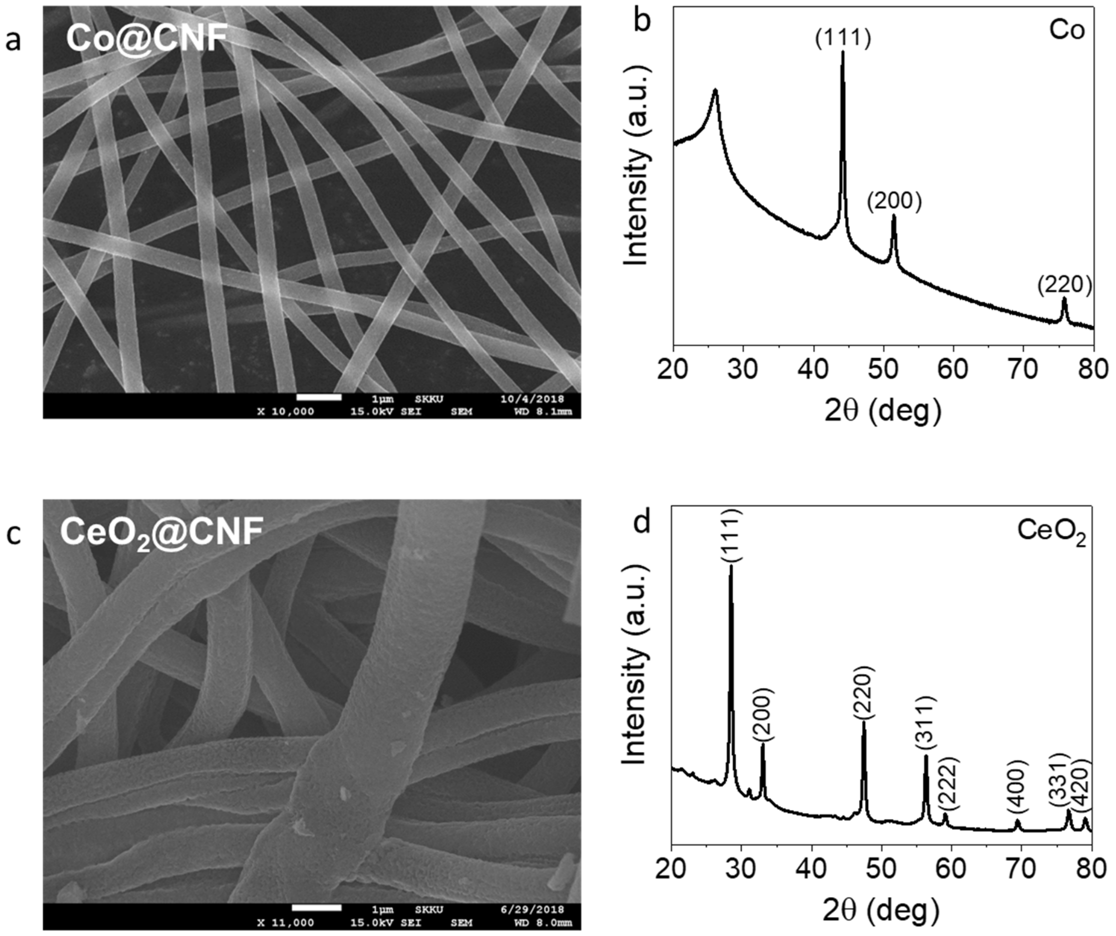

Figure S3. SEM images and XRD patterns of (**a, b**) Co@CNF and (**c, d**) CeO_2@CNF, respectively.

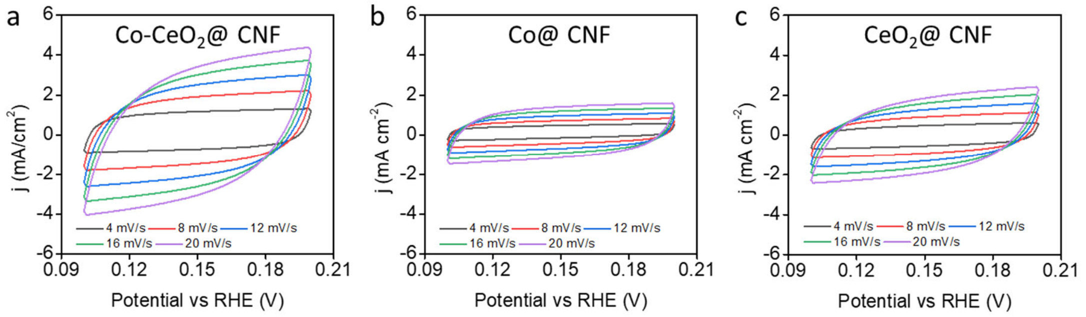

Figure S4. CV curves of (**a**)Co-CeO_2@CNF, (**b**)Co@CNF, and (**c**)CeO_2@CNF at various scan rates.

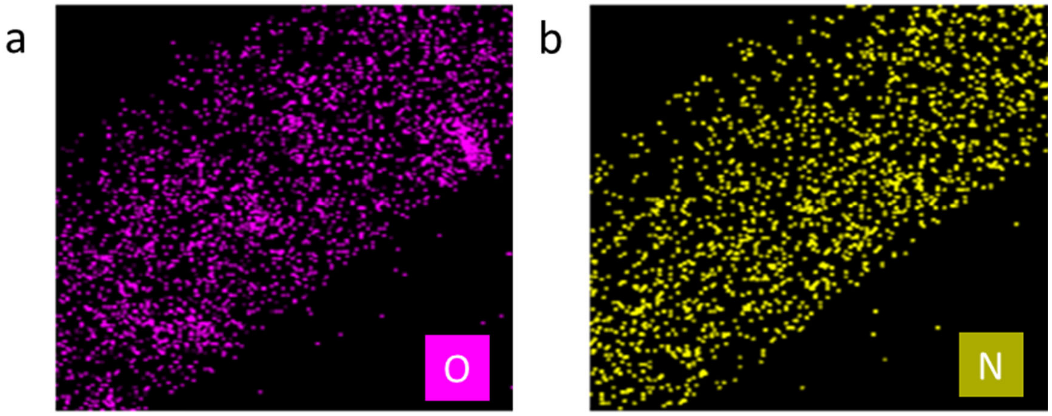

Figure S5. EDS elemental mapping TEM images of the Ni2Fe@CNF representing as (**a**) O and (**b**) N.

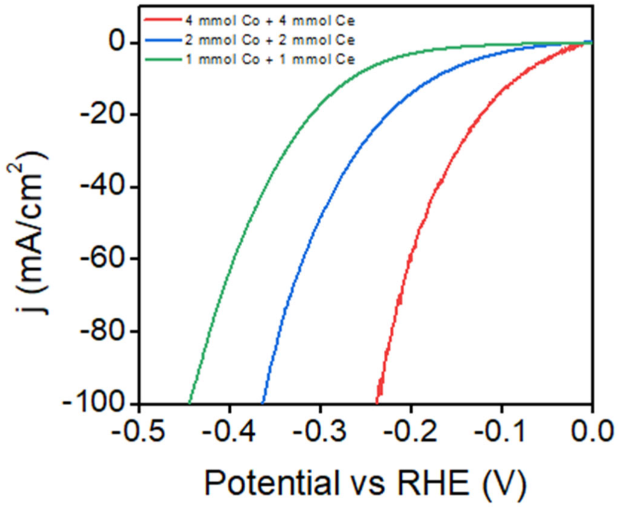

Figure S6. Polarization curves of Co-CeO_2@CNF at various amount of Co-CeO_2.

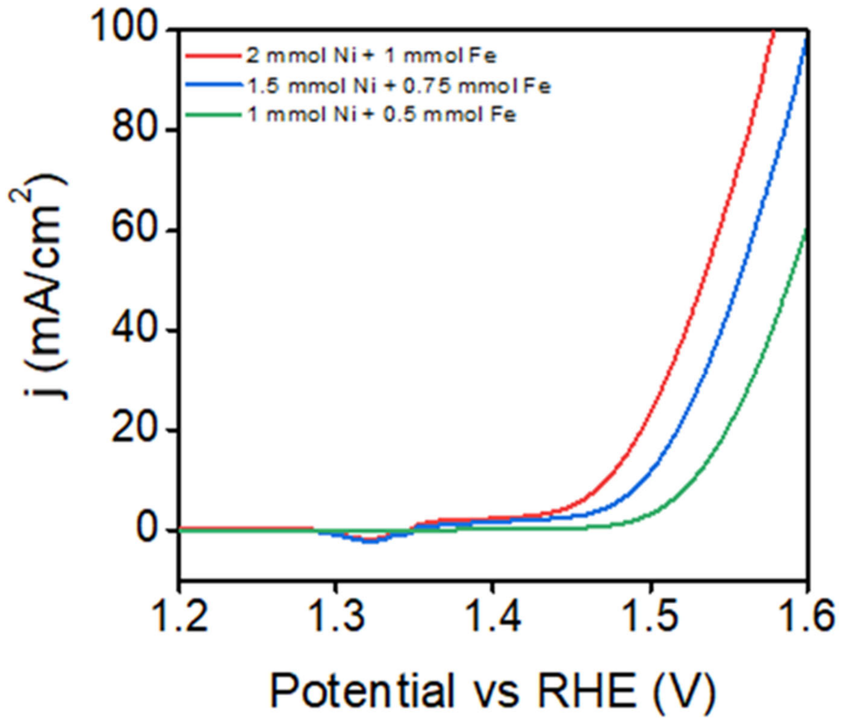

Figure S7. Polarization curves of Ni_2Fe@CNF at various amount of Ni_2Fe.

Table 1. HER performance compared to previously reported catalysts.

Catalysts	Electrolyte	HER overpotential vs RHE (Current Denstiy)	Reference
Co-CeO_2@CNF	1 M KOH	92 mV (−10 mA/cm^2)	This work
Co/Co_3O_4 core/ shell nanosheets	1 M KOH	129 mV (−20 mA/cm^2)	Nano Lett. 2015, 15, 6015
Fe0.5Co0.5@ NC/NCNS-800	1 M KOH	150 mV (−10 mA/cm^2)	J. Mater. Chem. A, 2017, 5, 5413–5425
Ni/ceria–rGO	1 M KOH	111 mV (−10 mA/cm^2)	J. Mater. Chem. A, 2017, 5, 8108–8116
3D-rGO-CeO_2	1 M KOH	340 mV (−10 mA/cm^2)	Eur. J. Inorg. Chem. 2018, 3952–3959
FeCoOOH/NF	1 M KOH	126 mV (−10 mA/cm^2)	Chem. Eur. J. 2018, 24, 1 – 6

Table 2. OER performance compared to previously reported catalysts.

Catalysts	Electrolyte	OER Overpotential Vs RHE (Current Denstiy)	Reference
Ni_2Fe@CNF	1 M KOH	242 mV (10 mA/cm^2)	This work
S-NiFe-700@C	1 M KOH	281 mV (10 mA/cm^2)	Sci. Rep. 2016, 6, 34004
HPGC@Ni_6Fe	1 M KOH	270 mV (10 mA/cm^2)	Nanoscale, 2017, 9, 11596-11604
S–FeNi@NC	1 M KOH	272 mV (20 mA/cm^2)	J. Mater. Chem. A, 2017, 5, 21320–21327
$Ni^{II}Fe^{II}$@NC	1 M KOH	239 mV (10 mA/cm^2)	Nano Energy, 2017, 39, 245–252
NiFe alloy	1 M KOH	298 mV (10 mA/cm^2)	Catal. Today, ASAP
